# Medicaid expansion and treatment for opioid use disorders in Oregon: an interrupted time-series analysis

**DOI:** 10.1186/s13722-019-0160-6

**Published:** 2019-08-15

**Authors:** Dennis McCarty, Yifan Gu, John W. McIlveen, Bonnie K. Lind

**Affiliations:** 1 OHSU/PSU School of Public Health, Oregon Health & Science University, 3181 SW Sam Jackson Park Road, Portland, OR 97239 USA; 2 Center for Health Systems Effectiveness, Oregon Health & Science University, 3181 SW Sam Jackson Park Road, Portland, OR 97239 USA; 3 Oregon Health Authority, State Opioid Treatment Authority, 500 Summer Street, Salem, OR 97301 USA

**Keywords:** Opioid use disorder, Opioid agonist therapy, Opioid antagonist therapy, Medicaid expansion, Psychosocial services for opioid use disorder, Medication for opioid use disorder

## Abstract

**Background:**

The study examined the association of the Affordable Care Act’s 2014 Medicaid expansion on the use of psychosocial services and pharmacotherapies for opioid use disorders among Oregon Medicaid recipients.

**Methods:**

Logistic regression analysis examined utilization of care before (January 1, 2010–December 31, 2013) and after Medicaid expansion in Oregon (January 1, 2014–December 31, 2016).

**Results:**

Adult membership in the Oregon Health Plan (Medicaid) increased 180% following Medicaid expansion (2013 = 172,539; 2014 = 482,081) and the number with a diagnosis of OUD nearly doubled (2013 = 6808; 2014 = 13,418). More individuals received psychosocial services (2013 = 4714; 2014 = 8781) and medications (2013 = 3464; 2014 = 6093) for opioid use disorder. The percent of patients receiving psychosocial services (69% to 65%) and the percent of individuals receiving pharmacotherapy (57% to 45%) declined primarily because of a decline in the proportion receiving care in an opioid treatment program (2013 = 41%; 2014 = 33%). Odds of accessing any psychosocial service increased by 8% per year from 2010 to 2013 (AOR = 1.08; 95% CI 1.06–1.11) with an 18% immediate decline associated with Medicaid expansion in 2014 (AOR = 0.82; 95% CI 0.76–0.87). Following Medicaid expansion, the odds of accessing psychosocial services increased 8% per year (2014 through 2016) (AOR = 1.08; 95% CI 1.06–1.11). Use of medications for opioid use disorder found no change in the odds of use in the years prior to Medicaid expansion, an immediate 36% (AOR = 0.64; 95% CI 0.60–0.68) decline in 2014, and a 13% increase per year in 2015 and 2016 (AOR = 1.13; 95% CI 1.09–1.16).

**Conclusion:**

The number of Medicaid recipients with an opioid use disorder who received psychosocial and pharmacological services increased substantially following Oregon’s Medicaid expansion in 2014. There was a decline, however, in the proportion of individuals with an opioid use disorder receiving care in opioid treatment programs.

**Electronic supplementary material:**

The online version of this article (10.1186/s13722-019-0160-6) contains supplementary material, which is available to authorized users.

## Background

The Affordable Care Act and its expansion of Medicaid eligibility promoted increased coverage for health care and improved access to treatment for alcohol, opioid, and other drug use disorders. Analyses of responses to the National Survey on Drug Use and Health reported reductions in people without insurance but no increase in the proportion who entered treatment for substance use disorders [1] and specifically for opioid use disorders [2]. An analysis of Medicaid Drug Utilization files for the years 2011 through 2014, however, observed quarterly increases in prescriptions for buprenorphine and Medicaid spending on buprenorphine; the increase overtime was substantially greater in expansion states [3]. A detailed look at which services individuals receive may shed light on how Medicaid expansion influenced access to care for opioid use disorders.

The 2014 Medicaid expansion reduced the uninsured rate in Oregon and facilitated potential access to treatment for people with opioid use disorders. In 2014, Oregon had elevated rates of (a) prescribing opioid analgesics (262 opioid prescription fills per 1000 residents), (b) opioid overdose hospitalizations (20.8 overdose hospitalizations per 1000 residents) and 7.1 overdose deaths per 100,000 residents and (c) overdose deaths (7.1 deaths per 1000 residents) (rates obtained from the Oregon Opioid Dashboard) [4]. Fentanyl first appeared in overdose deaths in 2014 with one death and climbed incrementally in 2015 (4 deaths), 2016 (12 deaths) and 2017 (49 deaths) [4]. An analysis of Oregon’s Medicaid data examined utilization of psychosocial services for opioid use disorders and medications for opioid use disorders. Analyses assessed change overtime in access to treatments for opioid use disorders on both the number in care and the relative distribution of where care occurred.

## Methods

### Data sources

De-identified Medicaid enrollment, claims and encounter data covering 4 years prior to Medicaid expansion (January 1, 2010 to December 31, 3013) and 3 years post Medicaid expansion (January 1, 2014 to December 31, 2016) were obtained from the Oregon Health Authority under a data use agreement. The Oregon Health and Science University’s Institutional Review Board reviewed the study protocol and approved study methods and human subject protections.

### Study population

The analysis was limited to adults with opioid use disorder (identified using ICD-9 and ICD-10 diagnostic codes) who were 18 to 64 years of age and continuously enrolled for at least 11 months in each study year. Individuals who were enrolled in both Medicare and Medicaid were excluded from the analysis because the Medicare data were not included in the data set. Oregon’s Medicaid program relies on 15 regional Medicaid managed care plans for 95% of its beneficiaries. Individuals not enrolled in a regional Medicaid managed care plan were excluded from the analysis.

### Outcome measures

Study outcomes assessed the use of psychosocial services for opioid use disorder (i.e., opioid treatment programs, counseling in specialty outpatient addiction treatment centers, residential and detoxification care in specialty addiction treatment centers and counseling in primary care settings), and the use of medications for opioid use disorder (i.e., buprenorphine, methadone, naltrexone and extended-release naltrexone). Counts of patients receiving one or more of the psychosocial services and medications for opioid use disorder were summed to assess total access to “any psychological services” and “any pharmacotherapy.”

ICD-9 and ICD-10 codes identified members with a diagnosed opioid use disorder (OUD). See Additional file 1: Table S1 for specific codes. Procedure codes (Current Procedural Terminology [CPT] Codes and Healthcare Common Procedural Code System), revenue codes, place of service codes, and pharmacy codes specified the use of opioid agonist therapy and other types of treatment received for opioid use disorder. Place of service codes differentiated care provided in primary care settings, outpatient specialty addiction treatment centers, and residential and detoxification services in specialty addiction treatment programs. Only claims that included both a psychosocial service and an OUD diagnosis were included in the count to avoid including psychosocial services received for reasons unrelated to OUD. Pharmacy claims identified individuals with prescriptions for buprenorphine, oral naltrexone and extended-release naltrexone. CPT codes identified individuals receiving extended-release naltrexone (because health plans tend to cover injectable medications as a medical procedure rather than a pharmacy claim) and methadone for opioid use disorder (because methadone cannot be prescribed for use as an opioid agonist therapy).

Federal regulations require opioid treatment programs (treatment centers approved to dispense methadone) to provide psychosocial services as well as opioid agonist therapy. Individuals enrolled in opioid treatment programs, therefore, were included in both the counts of any pharmacotherapy and any psychosocial service. Opioid treatment program services were billed at a bundled rate that covered both medication and psychosocial costs. Specific counseling visits and the type of medication, therefore, were not differentiated in the dataset. Most individuals receiving care in opioid treatment programs receive methadone and all were counted as receiving methadone. See Additional file 1: Table S1 for specific codes.

### Independent variables and covariates

Analytic models included adjustment for study year (coded 1 to 7), post-expansion (coded 0 in years 2010 to 2013 and 1 in years 2014 to 2016), and number of years post expansion (coded 0 in years 2010 to 2013 and sequentially 1 to 3 for years 2014 to 2016). Five covariates assessed associations with patient characteristics: age, gender, race/ethnicity (i.e., African American, American Indian/Alaskan Native, Asian/Pacific Islander, Hispanic/Latino, White, and unknown), residency (urban versus rural), and the presence of co-occurring psychiatric diagnoses (yes versus no).

### Analysis

Data were aggregated by calendar year. The unit of analysis was the person year. Logistic regression analyses assessed the association between Medicaid expansion, patient characteristics and the receipt of psychosocial services and medications for opioid use disorder. To access the impact of Medicaid expansion, the interrupted time series analysis [5] included the three measures of time: (1) study year (to control for secular trends), (2) Medicaid expansion (to test for immediate effects of expansion) and (3) years post Medicaid expansion (to test for continuing change and change in slope post expansion). A sensitivity analysis removed opioid treatment programs from the count of any psychosocial services to understand how inclusion of opioid treatment programs affected the proportion of individuals who received psychosocial services. Standard errors were clustered at the individual level to address the correlation between the longitudinal observations for members with multiple observations. Data management and analysis used R version 3.5.1 software.

## Results

### Numbers diagnosed with opioid use disorder

Medicaid expansion was associated with a 1.8-fold increase in adults enrolled in Oregon Medicaid comparing 2013 (*n *=172,539) to 2014 (*n *= 482,081). The number of individuals with a diagnostic indicator of an opioid use disorder nearly doubled (2013 = 6808; 2014 = 13,418) following Medicaid expansion and increased to more than 15,000 in 2015 (*n *= 15,251) and 2016 (*n *= 15,021). Table 1 summarizes the numbers and characteristics of the Medicaid enrollees with a diagnosis of opioid use disorder by year (2010–2016).

**Table 1:** Characteristics of Medicaid recipients with an OUD diagnosis

	2010		2011		2012		2013		2014		2015		2016	
	N	%	N	%	N	%	N	%	N	%	N	%	N	%
OUD population	3653	100	5733	100	6235	100	6808	100	13,418	100	15,251	100	15,021	100
Age (mean)	40.8	n/a	39.5	n/a	39.2	n/a	39.3	n/a	37.9	n/a	38.5	n/a	38.9	n/a
18–24 years	332	9.1	598	10.4	652	10.5	724	10.6	1556	11.6	1501	9.8	1248	8.3
25–34 years	1007	27.6	1819	31.7	2049	32.9	2164	31.8	4864	36.2	5439	35.7	5405	36.0
35–44 years	792	21.7	1239	21.6	1350	21.7	1575	23.1	3006	22.4	3520	23.1	3618	24.1
45–54 years	891	24.4	1246	21.7	1291	20.7	1299	19.1	2315	17.3	2670	17.5	2538	16.9
55–64 years	631	17.3	831	14.5	893	14.3	1046	15.4	1677	12.5	2121	13.9	2212	14.7
Gender														
Male	1173	32.1	2085	36.4	2245	36.0	2389	35.1	6245	46.5	7144	46.8	6860	45.7
Race/ethnicity														
White	2994	82.0	4724	82.4	5175	83.0	5658	83.1	10,670	79.5	12,262	80.4	12,021	80.0
Hispanic	173	4.7	292	5.1	286	4.6	338	5.0	1090	8.1	1158	7.6	1081	7.2
African American	232	6.4	302	5.3	309	5.0	340	5.0	495	3.7	551	3.6	552	3.7
American Indian/Alaskan Native	130	3.6	192	3.3	218	3.5	238	3.5	477	3.6	506	3.3	520	3.5
Asian/Pacific Islander	17	0.5	40	0.7	54	0.9	48	0.7	125	0.9	146	1.0	136	0.9
Other/unknown	107	2.9	183	3.2	193	3.1	186	2.7	561	4.2	628	4.1	711	4.7
Geography														
Rural	968	26.5	1505	26.3	1691	27.1	1957	28.7	3875	28.9	4591	30.1	4661	31.0
Psychiatric disorder	2545	69.7	3926	68.5	4156	66.7	4452	65.4	7536	56.2	8658	56.8	8723	58.1

Assessed among continuously enrolled adults (18–64) with an OUD diagnosis, not dually eligible for Medicaid and Medicare, and enrolled in a regional Medicaid health plan

### Counts of psychosocial services

**Fig. 1:**
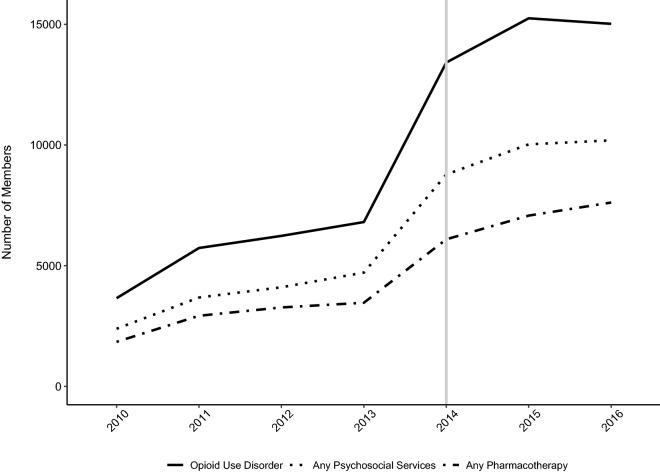
Change in numbers with an opioid use disorder, receiving psychosocial services and/or medications for opioid use disorders (2010 to 2016)

The number of Medicaid recipients receiving any psychosocial service for opioid use disorder increased 86% following Medicaid expansion (2013 = 4714; 2014 = 8781) and increased to more than 10,000 in 2015 (*n *= 10,028) and 2016 (*n *= 10,193). Increased access was observed in all psychosocial service settings between 2013 and 2014: specialty outpatient (*n *= 2106 vs. *n *= 3974), specialty residential (*n *= 675 vs. *n *= 1787), primary care (*n *= 125 vs. *n *= 478), and care in opioid treatment programs (*n *=2786 vs. *n *= 4394). Table 2 details the annual counts and Fig. 1 plots the change over time.

**Table 2:** Counts and percentages of Medicaid recipients with OUD who received psychosocial services and/or pharmacotherapy

	2010	2011	2012	2013	2014	2015	2016
Total study population (N)	107,398	158,677	167,939	172,539	482,081	491,125	455,607
Opioid use disorder (N, %)							
OUD diagnosis	3653 (3.4)	5733 (3.6)	6235 (3.7)	6808 (4.0)	13,418 (2.8)	15,251 (3.1)	15,021 (3.3)
Psychosocial services (N, %)							
Residential and detoxification	83 (2.3)	221 (3.9)	414 (6.6)	675 (9.9)	1787 (13.3)	1844 (12.1)	2078 (13.8)
Opioid treatment program	1651 (45.2)	2555 (44.6)	2747 (44.1)	2786 (40.9)	4394 (32.8)	4694 (30.8)	4683 (31.2)
Primary care	123 (3.4)	81 (1.4)	98 (1.6)	125 (1.8)	478 (3.6)	594 (3.9)	938 (6.2)
Specialty outpatient	930 (25.5)	1460 (25.5)	1620 (26.0)	2106 (30.9)	3974 (29.6)	4898 (32.1)	4882 (32.5)
Any psychosocial services	2386 (65.3)	3682 (64.2)	4106 (65.9)	4714 (69.2)	8781 (65.4)	10,028 (65.8)	10,193 (67.9)
Pharmacotherapy (N, %)							
Buprenorphine	206 (5.6)	396 (6.9)	511 (8.2)	634 (9.3)	1483 (11.1)	2006 (13.2)	2335 (15.5)
Methadone	1651 (45.2)	2555 (44.6)	2747 (44.1)	2786 (40.9)	4394 (32.8)	4694 (30.8)	4683 (31.2)
Oral naltrexone	4 (0.1)	13 (0.2)	25 (0.4)	38 (0.6)	83 (0.6)	173 (1.1)	304 (2.0)
Naltrexone ext-release	2 (0.1)	1 (0.0)	51 (0.8)	134 (2.0)	459 (3.4)	711 (4.7)	941 (6.3)
Any pharmacotherapy	1844 (50.5)	2925 (51.0)	3270 (52.5)	3464 (50.9)	6093 (45.4)	7075 (46.4)	7617 (50.7)

### Counts of pharmacotherapy

More individuals with opioid use disorder received pharmacotherapy with a medication for opioid use disorder between 2013 and 2014: buprenorphine (*n *= 634 vs. *n *= 1483), methadone (*n *= 2786 vs. *n *= 4394), extended-release naltrexone (*n *= 134 vs. *n *= 459) and oral naltrexone (*n *= 38 vs. *n *= 83). Access to pharmacotherapy for opioid use disorder improved to more than 7000 individuals in 2015 (*n *= 7075) and 2016 (*n *= 7617). See Table 2 and Fig. 1.

### Proportion using psychosocial services

The proportion of individuals with a diagnostic indication of an opioid use disorder receiving any psychosocial services for opioid use disorder increased to 69% in 2013 and declined to 65% in 2014 with subsequent increases to 66% (2015) and 68% (2016). The proportion in specialty outpatient care grew from 26% (2010–2012) to 30% (2013–2014) to 32% (2015–2016).

**Fig. 2:**
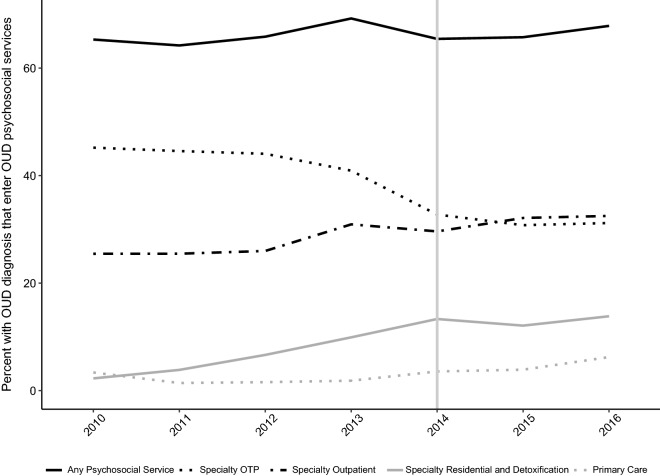
Change in proportion of individuals with opioid use disorders receiving psychosocial services (2010 to 2016)

Logistic regression analyses assessed change over the study period and tested the impact of Medicaid expansion. Odds of any psychosocial service use increased 8% (AOR = 1.08, 95% CI 1.06–1.11) per year over the study period with an 18% immediate decline (AOR = 0.82, 95% CI 0.76–0.87) associated with Medicaid expansion and a nonsignificant change to the overall slope in 2015 and 2016. The adjusted odds ratios for the covariates suggested that men were less likely than women to receive psychosocial services, urban residents were more likely to receive care, and people with psychiatric diagnoses were less likely to enter psychosocial services. Table 3 summarizes the logistic regression analyses. The unadjusted trends over time for access to psychosocial services are plotted in Fig. 2. Patterns varied but the general trend was an increase over time with a dip at Medicaid expansion. The 2014 decline into the proportion receiving any psychosocial service appears to be related to the 2013 to 2014 decrease in the relative use of opioid treatment programs. The sensitivity analysis found the use of psychosocial services increased year by year over the study period when services in opioid treatment programs were removed from the analysis. See Additional file 1: Table S2.

**Table 3:** Multi-variable logistic regression analysis of the use of any psychosocial services or any pharmacotherapy among people with OUD

	Any psychosocial services		Any pharmacotherapy	
	Adjusted- odds ratio	95% CI	Adjusted-odds ratio	95% CI
Year	1.08	(1.06, 1.01)	1.01	(0.99, 1.03)
Post expansion (reference pre-expansion)	0.82	(0.76, 0.64)	0.64	(0.60, 0.68)
Year after expansion	0.98	(0.95, 1.13)	1.13	(1.09, 1.16)
Age	1.01	(1.01, 1.00)	1.00	(1.00, 1.00)
Gender: male (reference female)	0.93	(0.89, 0.94)	0.94	(0.89, 0.98)
Residence: urban (reference rural)	1.97	(1.88, 2.57)	2.57	(2.44, 2.71)
Race: African American	0.95	(0.84, 1.06)	1.01	(0.89, 1.14)
Race: American Indian/Alaskan Native	0.90	(0.80, 1.01)	0.84	(0.73, 0.96)
Race: Asian/Pacific Islander	0.91	(0.72, 1.15)	0.94	(0.73, 1.20)
Race: hispanic	0.95	(0.87, 1.04)	0.96	(0.88, 1.06)
Race: other/unknown	0.87	(0.79, 0.97)	0.91	(0.81, 1.03)
Race: white (reference) Psychiatric disorder: yes (reference no)	0.84	(0.81, 0.87)	0.63	(0.60, 0.65)

“Year” is coded 1 to 7; “Post expansion” is coded 0 for years 2010 to 2013, and 1 for years 2014 to 2016; “Year after expansion” is coded 0 for years 2010 to 2013, and 1, 2, 3 for years 2014 to 2016; “Age” is in years; the reference level of gender is female; the reference level of “Residence” is rural; the reference level of “Race” is White; the reference level of “Psychiatric Disorder” is “no psychiatric disorder diagnosis”

### Proportion using a medication for opioid use disorder

Despite the increase in the number of Medicaid recipients receiving opioid agonist or opioid antagonist therapy, the proportion of members with a diagnostic indication of an opioid use disorder using a medication for opioid use disorder declined following Medicaid expansion (2013 = 51%; 2014 = 45%). See Table 2.

**Fig. 3:**
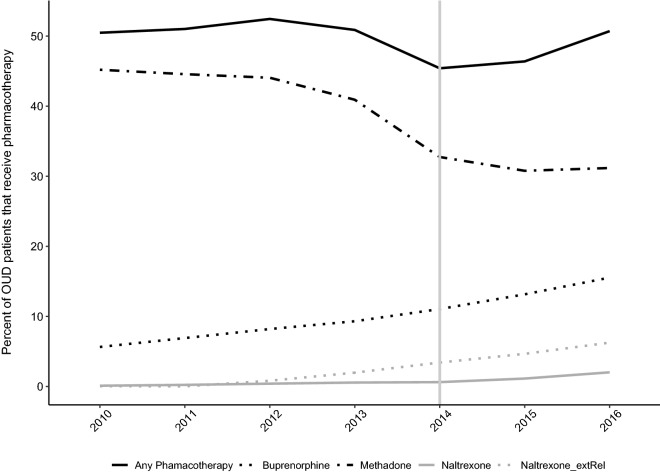
Change in proportion of individuals with opioid use disorders receiving medications for opioid use disorders (2010 to 2016)

In the multi-variable logistic regression analysis of use of any pharmacotherapy, odds of use were unchanged in the years prior to expansion (AOR = 1.01, 95% CI 0.99–1.03). Medicaid expansion, however, was associated with an immediate 36% decline in the likelihood of using medication (AOR = 0.64, 95% CI 0.60–0.68). Following the immediate decline, the odds of any pharmacotherapy increased by 13% per year in 2015 and 2016 (AOR = 1.13, 95% CI 1.09–1.16). In addition, men, American Indians, Asians or Pacific islanders, and Hispanic (compared to White) and individuals with psychiatric disorders were less likely to receive medication while urban residents were more likely to receive a medication for opioid use disorder. See Table 3 for the logistic regression results. Overall, there was a steady decline in the proportion of individuals receiving methadone and an increase in the use of buprenorphine and extended-release naltrexone. Unadjusted trends over time are displayed in Fig. 3. See Supplemental Table 3 for the regression analyses on buprenorphine and methadone. Regression analyses for naltrexone use were not conducted because counts were too small for stable models.

## Discussion

Oregon’s Medicaid expansion was associated with a nearly twofold increase in the number of individuals enrolled in Medicaid health plans, the number of individuals with a diagnosis indicating an opioid use disorder, receiving psychosocial services, and receiving an opioid agonist or an opioid antagonist medication to support recovery from opioid use disorder. The overall increase in Medicaid recipients receiving care for opioid use disorder illustrates the value of Medicaid expansion and its role in addressing the opioid epidemic.

The decline in the proportion of Medicaid recipients receiving care in opioid treatment programs from 45% (2010) to 31% (2016) reflects a long-term change in the use of methadone. Increased use of buprenorphine (2010 = 6%; 2016 = 16%) and naltrexone (2010 = 0%; 2016 = 8%) offset the decline in the use of methadone and, overall, the rate of individuals using opioid agonist or antagonist medications was stable at about 50%. The relatively abrupt decline in the proportion enrolled in opioid treatment programs between 2013 (41%) to 2014 (33%) could reflect greater enrollment in Medicaid from rural communities or increased wait lists within opioid treatment programs. The proportion of Medicaid recipients with an opioid use disorder from rural zip codes, however, was unchanged at 29% in 2013 and 2014 (see Table 1). The State Opioid Treatment Authority does not maintain a wait list and believes that in most areas of the state admissions to opioid treatment programs occur within days of seeking care. Oregon’s Medicaid Managed Care plans require prior authorization for medications for opioid use disorder and some may require individuals to fail first at less expensive therapies; these policies may have contributed to the proportional decline in the use of opioid treatment programs. Another possibility is that the individuals covered under Medicaid expansion may have been new to care and reluctant to initiate care within an opioid treatment program.

Medicaid expansion was also associated with increases in the proportion receiving residential detoxification and post-detoxification care, and a small increase in the proportion receiving counseling services in primary care settings. The distributional shifts may reflect patient preferences, improved choice and the difficulty of matching patients to appropriate levels of care.

Medicaid recipients in rural communities, people with a psychiatric disorder, and men were less likely to access psychosocial and opioid agonist and antagonist medications for opioid use disorder. American Indians were also less likely to access opioid agonist and antagonist medications. Geography and long travel times inhibit access to care in rural communities. Oregon’s American Indian tribes have been reluctant to use opioid agonist medication and efforts to facilitate use of medication continue in tribal settings. Historically, Oregon’s specialty addiction treatment services have not been licensed to treat mental health disorders and this may account for the reduced access among individuals with psychiatric disorders.

## Limitations

The analysis is limited because the data were from a single state in the northwest region of the United States. Generalizability is uncertain to other states and regions. Substance use disorders are frequently under diagnosed and, especially in 2014, underdiagnoses within the Medicaid expansion population may have distorted the proportions receiving care. The proportional decline in the use of methadone and the increased use of buprenorphine are under-estimated because the data do not break out the use of specific medications in opioid treatment programs. The ICD-9 and ICD-10 diagnostic codes used to identify patients with opioid use disorder assess opioid dependence rather than the current American Psychiatric Association’s definition of opioid use disorder [6]. Finally, the study is a retrospective cohort analysis that cannot establish causation.

## Conclusion

Oregon’s 2014 Medicaid expansion was associated with increased access to and utilization of psychosocial services for opioid use disorders and use of medications for opioid use disorder. The numbers accessing care doubled and reflected changes in the services provided to treat opioid use disorders.

## Additional file

**Additional file 1.** Additional tables.

## Data Availability

A data use agreement with the Oregon Health Authority authorized the use of the data for this study and does not permit the data set to be used for other analyses.

## Abbreviations

AOR: adjusted odds ratio
CI: confidence interval
CPT: current procedural terminology
ICD: International Classification of Diseases
OUD: opioid use disorder
