# Supplementary material for: Medicaid expansion and treatment for opioid use disorders in Oregon: an interrupted time-series analysis
Source: Addict Sci Clin Pract. 2019 Aug 15;14:31. doi: 10.1186/s13722-019-0160-6 (PMC6694675; doi:10.1186/s13722-019-0160-6)
Supplement: Supplementary file 1 — Additional file 1. Additional tables. [file 13722_2019_160_MOESM1_ESM.docx]

**Additional Table S1. Codes Used in the Analysis**

**ICD 9 codes used for identifying opioid use disorder diagnosis**

- 30400 30401 30402 30470 30471 30472 30550 30551 30552

**ICD10 codes used for identifying opioids use disorder diagnosis**

- F1110, F11120, F11121, F11122, F11129, F1114, F11150, F11151, F11159, F11181, F11182, F11188, F1119, F1120, F11220, F11221, F11222, F11229, F1123, F1124, F11250, F11251, F11259, F11281, F11282, F11288, F1129

**Codes used for identifying outpatient, residential care, and detoxification**

Acute Inpatient (Residential care):

- CPT: 99221, 99222, 99223, 99231, 99232, 99233, 99238, 99239, 99251, 99252, 99253, 99254, 99255, 99291
- Revenue code: 0100, 0101, 0110, 0111, 0112, 0113, 0114, 0119, 0120, 0121, 0122, 0123, 0124, 0129, 0130, 0131, 0132, 0133, 0134, 0139, 0140, 0141, 0142, 0143, 0144, 0149, 0150, 0151, 0152, 0153, 0154, 0159, 0160, 0164, 0167, 0169, 0200, 0201, 0202, 0203, 0204, 0206, 0207, 0208, 0209, 0210, 0211, 0212, 0213, 0214, 0219, 0720, 0721, 0722, 0723, 0724, 0729, 0987

Outpatient:

- CPT: 99201, 99202, 99203, 99204, 99205, 99211, 99212, 99213, 99214, 99215, 99241, 99242, 99243, 99244, 99245, 99341, 99342, 99343, 99344, 99345, 99347, 99348, 99349, 99350, 99381, 99382, 99383, 99384, 99385, 99386, 99387, 99391, 99392, 99393, 99394, 99395, 99396, 99397, 99401, 99402, 99403, 99404, 99411, 99412, 99420, 99429, 99455, 99456, G0402, G0438, G0439, G0463, T1015
- Revenue code: 0510, 0511, 0512, 0513, 0514, 0515, 0516, 0517, 0519, 0520, 0521, 0522, 0523, 0526, 0527, 0528, 0529, 0982, 0983

Detoxification:

- CPT: H0008, H0009, H0010, H0011, H0012, H0013, H0014
- HCPCS: 9462, 9465, 9468
- Revenue code: 0116, 0126, 0136, 0146, 0156

**Codes for identifying specialty outpatient, specialty residential and detoxification care, primary care and opioid treatment program**

We combined the indicators for outpatient care, acute inpatient and detoxification with place of services (pos) code to determine the categorization of the treatment services:

- Specialty Outpatient: Outpatient + pos = 11,12,15, 53,57,71
- Specialty Residential and Detoxification Care: Acute Inpatient + pos=11,55,61; or Detoxification + pos=11,53,55; or pos = 55
- Primary Care: outpatient + pos=20,50,72
- Opioid treatment program (Methadone): cpt = H0020, J1230

**Codes for Pharmacotherapy:**

**Naltrexone NDC:**

"00406009201" "00406009203" "00185003901" "00185003930" "00406117001" "00406117003" "00555090201" "00555090202" "16729008101" "16729008110" "42291063230" "43063059115" "47335032683" "47335032688" "50436010501" "51224020630" "51224020650" "52152010502" "52152010504" "52152010530" "54868557400" "65694010003" "65694010010" "68084029111" "68084029121" "68094085362" "68115068030" "00056001122" "00056001130" "00056001170" "00056007950" "51285027501" "51285027502"

**Naltrexone (extended release) NDC:** "63459030042" "65757030001" "65757030202"

**Buprenorphine NDC:**

"63481016101" "63481016160" "63481020701" "63481020760" "63481034801" "63481034860" "63481051901" "63481051960" "63481068501" "63481068560" "63481082001" "63481082060" "63481095201" "63481095260" "00054017613" "00054017713" "00093537856" "00093537956" "00228315303" "00228315603" "00378092393" "00378092493" "35356055530" "35356055630" "50383092493" "50383093093" "55700030230" "55700030330" "68308020230" "68308020830" "12496127802" "12496131002" "49999063830" "49999063930" "63874117303"

**Buprenorphine with naltrexone NDC:**

"59385001201" "59385001230" "59385001401" "59385001430" "59385001601" "59385001630" "00054018813" "00054018913" "00093572056" "00093572156" "00228315403" "00228315473" "00228315503" "00228315573" "00406192303" "00406192403" "42291017430" "42291017530" "50383028793" "50383029493" "54569640800" "55700018430" "65162041503" "65162041603" "12496120201" "12496120203" "12496120401 "12496120403" "12496120801" "12496120803" "12496121201" "12496121203" "54569639900" "55700014730" "12496128302" "12496130602" "16590066630" "35356000407" "35356000430" "43063018407" "43063018430" "49999039507" "49999039515" "49999039530" "52959030430" "52959074930" "54569549600" "54569573900" "54569573901" "54569573902" "54868570700" "54868570701" "54868570702" "54868570703" "54868570704" "54868575000" "55045378403" "63629402801" "63629403401" "63629403402" "63629403403" "63874108403" "63874108503" "66336001630" "68071138003" "68071151003" "68258299903" "54123011430" "54123091430" "54123092930" "54123095730" "54123098630"

**Methadone CPT:** "H0020" "J1230" **Vivitrol CPT:** "J2315" "T1502"

**Additional Table S2. Sensitivity analysis - Logistic regression analysis of psychosocial services excluding OTP ^[[1]](#footnote-1)^**

| Variables | Any Psychosocial Services excluding OTP | | |
| --- | --- | --- | --- |
|  | AOR^[[2]](#footnote-2)^ | 95% CI | |
| Year | 1.21 | 1.17 | 1.24 |
| Post expansion | 1.13 | 1.06 | 1.20 |
| Year after expansion | 0.90 | 0.87 | 0.93 |
| Age | 1.00 | 1.00 | 1.00 |
| Gender(Male) | 1.05 | 1.01 | 1.09 |
| Urban | 0.84 | 0.81 | 0.88 |
| Race (African American) | 0.82 | 0.74 | 0.92 |
| Race (American Indian/Alaskan Native) | 0.89 | 0.80 | 0.99 |
| Race (Asian/Pacific Islander) | 0.97 | 0.78 | 1.22 |
| Race (Hispanic) | 0.95 | 0.88 | 1.03 |
| Race (Other/Unknown) | 0.91 | 0.83 | 1.00 |
| Psychiatric Disorder | 1.71 | 1.65 | 1.78 |

**Additional Table S3. Logistic regression analysis of psychosocial services and buprenorphine and methadone ^[[3]](#footnote-3)^**

| Variables | Specialty Outpatient | | | Residential & Detoxification | | | Primary Care | | | Buprenorphine | | | Methadone | | |
| --- | --- | --- | --- | --- | --- | --- | --- | --- | --- | --- | --- | --- | --- | --- | --- |
|  | AOR^[[4]](#footnote-4)^ | 95% CI | | AOR | 95% CI | | AOR | 95% CI | | AOR | 95% CI | | AOR | 95% CI | |
| Year | 1.11 | 1.08 | 1.15 | 1.67 | 1.58 | 1.78 | 0.85 | 0.77 | 0.93 | 1.18 | 1.13 | 1.24 | 0.94 | 0.92 | 0.97 |
| Post expansion | 1.03 | 0.96 | 1.10 | 1.18 | 1.07 | 1.31 | 1.72 | 1.37 | 2.16 | 0.98 | 0.89 | 1.07 | 0.64 | 0.60 | 0.68 |
| Year after expansion | 0.95 | 0.91 | 0.98 | 0.63 | 0.59 | 0.67 | 1.61 | 1.44 | 1.81 | 1.04 | 0.98 | 1.10 | 1.03 | 1.00 | 1.07 |
| Age | 1.01 | 1.01 | 1.02 | 0.95 | 0.95 | 0.95 | 1.00 | 1.00 | 1.00 | 0.98 | 0.97 | 0.98 | 1.01 | 1.01 | 1.01 |
| Gender(Male) | 0.98 | 0.94 | 1.02 | 1.33 | 1.25 | 1.41 | 0.89 | 0.80 | 0.99 | 1.00 | 0.93 | 1.08 | 0.93 | 0.88 | 0.99 |
| Urban | 0.81 | 0.78 | 0.85 | 1.30 | 1.22 | 1.38 | 0.76 | 0.69 | 0.85 | 0.60 | 0.56 | 0.64 | 4.51 | 4.18 | 4.86 |
| Race (African American) | 0.80 | 0.71 | 0.89 | 1.04 | 0.87 | 1.23 | 0.80 | 0.58 | 1.10 | 0.75 | 0.59 | 0.96 | 1.09 | 0.95 | 1.25 |
| Race (American Indian/Alaskan Native) | 0.86 | 0.77 | 0.97 | 1.24 | 1.07 | 1.44 | 0.70 | 0.51 | 0.95 | 0.66 | 0.53 | 0.83 | 0.99 | 0.85 | 1.17 |
| Race (Asian/Pacific Islander) | 1.04 | 0.82 | 1.33 | 1.04 | 0.77 | 1.41 | 0.66 | 0.34 | 1.28 | 1.36 | 0.93 | 1.96 | 0.81 | 0.60 | 1.10 |
| Race (Hispanic) | 0.93 | 0.86 | 1.02 | 1.04 | 0.93 | 1.17 | 0.92 | 0.75 | 1.12 | 0.81 | 0.70 | 0.94 | 1.04 | 0.93 | 1.17 |
| Race (Other/Unknown) | 0.89 | 0.80 | 1.00 | 1.00 | 0.87 | 1.15 | 0.93 | 0.71 | 1.21 | 1.01 | 0.86 | 1.19 | 0.87 | 0.75 | 1.01 |
| Psychiatric Disorder | 1.70 | 1.63 | 1.77 | 1.48 | 1.40 | 1.57 | 1.79 | 1.61 | 1.97 | 1.23 | 1.15 | 1.31 | 0.55 | 0.52 | 0.57 |

1. “Year” is coded 1 to 7; “Post expansion” is coded 0 for years 2010 to 2013, and 1 for years 2014 to 2016; “Year after expansion” is coded 0 for years 2010 to 2013, and 1, 2, 3 for years 2014 to 2016; “Age” is in years; the reference level of gender is female; the reference level of “Residence” is rural; the reference level of “Race” is White; the reference level of “Psychiatric Disorder” is “no psychiatric disorder diagnosis”. OTP refers to Opioid Treatment Program. [↑](#footnote-ref-1)
2. Adjusted odds ratio [↑](#footnote-ref-2)
3. “Year” is coded 1 to 7; “Post expansion” is coded 0 for years 2010 to 2013, and 1 for years 2014 to 2016; “Year after expansion” is coded 0 for years 2010 to 2013, and 1, 2, 3 for years 2014 to 2016; “Age” is in years; the reference level of gender is female; the reference level of “Residence” is rural; the reference level of “Race” is White; the reference level of “Psychiatric Disorder” is “no psychiatric disorder diagnosis”. [↑](#footnote-ref-3)
4. Adjusted odds ratio [↑](#footnote-ref-4)
